# Assessment of noise level and risk of hearing loss among *taiko* musicians

**DOI:** 10.47626/1679-4435-2020-591

**Published:** 2021-03-03

**Authors:** Vanessa Yumi Hirata, Gisele Dias Buss, José Fernando Polanski

**Affiliations:** 1 Otorrinolaringologia, Faculdade Evangélica Mackenzie do Paraná, Curitiba, PR, Brazil; 2 Otorrinolaringologia, Universidade Federal do Paraná, Curitiba, PR, Brazil

**Keywords:** music, noise, hearing loss

## Abstract

**Introduction:**

*taiko* music is performed with specific drums, which produce loud and low-tone sounds that can potentially lead to hearing risk.

**Objectives:**

To assess sound pressure levels and hearing safety of musicians who play in *taiko* groups.

**Methods:**

Using a decibel meter, noise exposure was measured in two different groups (group 1 and group 2), which are divided into categories: (group 1 = five categories - junior, free, general a, general b, and master, and group 2 = two categories - adult and child). The calculation of the daily noise dose was based on the Brazilian Occupational Hygiene Standard 01, which establishes the following classification: acceptable dose (between 0 and 50%), above the action level (50 to 80%), uncertain dose level (80 to 100%), and above the acceptable level (more than 100%).

**Results:**

In group 1 categories, the daily noise doses obtained were: junior = 88%; general B = 423%; master = 218%; general A = 370%, and free = 150%. In the adult and children categories of group 2, the results were 127 and 17%, respectively.

**Conclusions:**

*taiko* musicians are exposed to daily noise doses above the safe level, except for the junior categories in group 1, and the children, in group 2 - which showed daily noise doses at an uncertain dose level, and at an acceptable level, respectively.

## INTRODUCTION

Music has been present in people’s lives since ancient times, playing an important role in human communication,^1^ as well as in the expression of culture and origin of peoples. The *taiko*, or “big drum”, in Japanese, is a musical instrument that produces an intense and low-pitch sound that has its origins dating back to the excavations of the Joumon era (10 000-300 B.C.), which suggests that it was used in religious and ceremonial occasions in ancient Japan.^2^

Noise, in turn, is characterized by any exposure that exerts an average of 90 dB or more than 85 dB(A) - in an A-weighted circuit. This circuit is used in an occupational assessment with integrators carried by the evaluated person, for 8 hours a day, regularly, over a period of years.^3^ The definitions, technical recommendations, and restriction criteria related to noise exposure are defined by technical standards edited by government health and labor agencies, such as the Brazilian Ministry of Labor and the American National Institute for Occupational Safety and Health (NIOSH). Some examples of Brazilian standards are the Regulatory Standard 15 (NR-15)^4^ and the Occupational Hygiene Standard 01 (NHO-01),^5^ among others.

Exposure to noise is considered the second most common cause of sensorineural hearing loss and accounts for about 16% of the disabling hearing loss in the adult population worldwide. ^6^ Musicians, specifically due to the time of exposure to high-intensity sounds, may also be subject to hearing risks.^7-11^

The main noise-induced hearing changes occur in the organ of Corti, since prolonged exposure to intense sounds causes progressive destruction of the outer and inner hair cells, the former being more sensitive to high and prolonged sound pressures, suffering metabolic exhaustion, oxidative stress, ischemia, and cell death. The space left by the dying cells is filled with fibrotic scar tissue, which results in a permanent deficit in hearing ability.^12,13^ In the case of hearing loss, this harmful exposure is generally not painful or related to hearing discomfort,^14^ which can cause the individual to neglect the problem. In addition to hypoacusis, there may be tinnitus, dizziness, and a feeling of fullness in the ear. Still, changes in the cardiovascular, gastrointestinal, muscular, and nervous systems may occur - changes in mood, stress and irritability.^15,16^

Considering that *taiko* percussionists are exposed to noise and, consequently, to hearing risks, the relevance of this study is justified both by the social aspect (due to the large number of *taiko* musicians around the world - in Japan alone, it is estimated that there are 200 thousand adepts) and by the musicians’ hearing health and general health. In addition, hearing loss related to intense noise exposure is known to be a cumulative and insidiously progressive, irreversible and chronically progressive condition, but one that can be prevented.^12^

Furthermore, as far as this research has been able to find, there are no scientific publications involving *taiko*. The works found in the main databases focus on the acoustics recommended for Western classical musical performances, including organ, orchestral, opera, and, in a few cases, rock concerts.^17^ Thus, this study aimed to measure the intensity of the sounds generated by *taiko* drums and to verify the hearing safety of the musicians, according to the definitions of the NHO 01 for the assessment of noise exposure.

## METHODS

A cross-sectional observational study was conducted with 2 groups of *taiko*, which was approved by the Research Ethics Committee of the institution under approval number 2.722.581.

Group 1 comprised 60 members divided into 5 categories: Junior (from 6 to 18 years old), Master (over 40 years old), General A, General B, and Free, the last 3 separated by criteria for participation in competitions. During the rehearsals, the group used odaiko, *nagadô, okedô, hiradaiko, shimejishi, kanê, tekkan, and shimê* drum models. Group 2, in turn, included 150 members divided into 2 categories - child and adult -, using the *odaiko eisã* (small, medium, and large), paranku, and shimê models. Each category was considered a homogeneous group composed of individuals of both sexes and age range from 6 to 65 years. Each group is considered to have a similar exposure among its participants.^5^

The measurements were made in rehearsals conducted from July 2018 to February 2019. The rehearsals were measured in full, in the usual environment of the studied groups, since the set of measurements must be representative of the real conditions of occupational exposure of participants in the exercise of their functions. ^5^ For the measurements, we used the THDL-400 calibrated decibel meter (Instrutherm, São Paulo, Brazil). The device met the specifications contained in the International Electrotechnical Commission (IEC) 804.

To assess auditory risk, 85 dB(A) was used as a reference criterion, which corresponds to a dose of 100% for a safe exposure of 8 hours, and dose doubling increase of 3 (q = 3), in which the increase of 3 dB(A) implies a reduction of exposure to half of the maximum allowed time (Table 1). This is the safety parameter established by the NHO 01, which determines criteria and procedures for assessing occupational noise exposure that imply a potential risk of deafness.

**Table 1:** Maximum allowable daily exposure time according to the level of noise

Level of noise [dB(A)]	Maximum allowable daily time (Tn)
85	480.00
86	380.97
87	302.38
88	240.00
89	190.48
90	151.19
91	120.00
92	95.24
93	75.59
94	60.00
95	47.62
96	37.79
97	30.00
98	23.81
99	18.89
100	15.00
101	11.9
102	9.44
103	7.5
104	5.95
105	4.72
106	3.75
107	2.97
108	2.36
109	1.87
110	1.48

Source: adapted from the Brazilian Occupational Hygiene Standard NHO 01.^5^

dB(A) = decibels in A-weighted circuit; Tn = maximum daily time in minutes allowable at that level, according to the NHO 01.

The technical specifications mentioned above apply to the noise of the drums, which is of the continuous or intermittent type, meaning that it does not exceed the exposure limit of 115 dB(A). The decibel meter was positioned at a distance of 1 meter from the musicians, since the NHO 01 recommends that the assessment procedures should interfere as little as possible in the environmental and operational conditions characteristic of the work condition under study.

To compile the data, all rehearsals were filmed with a focus on the decibel meter display. As there was a large fluctuation of dB (A) values in a short period of time, it was not possible to measure the sound intensity in real time. Therefore, we used the Media Player Classic Home - edition 64, with *slow motion* function (speed: 0.13x), so that the number of times each recorded dB(A) appeared - in the range of 85 to 110 dB(A) - could me manually computed. The mean time of change in the dB(A) value was of 0.304 seconds, which corresponds to approximately 3 dB(A) variations per second. Next, the exposure time for each dB(A) in minutes was calculated, which was used as a numerator in the calculation of occupational noise exposure (daily dose).

The calculation of the daily dose of NHO 01 refers to an 8-hour working day. However, *taiko* presentations and rehearsals last less than a working period, and even if the rehearsals last for hours, noise exposure does not represent the total rehearsal time - which influences the results obtained with the daily dose calculation. As there are no publications of a pre-defined method for this evaluation, the calculation was conducted in an adapted manner, proportional to the duration of the performances, according to the following expression:

**Table:** 

DAILY DOSE = C_1_/T_1_ + C_2_/T_2_ + C_3_/T_3_ + ... + C_n_/T_n_ . (%)

where C_n_ is the total daily time in minutes that the musician is exposed to a noise level of the drums, T_n_ is the maximum daily time in minutes allowable at that level, according to Table 1 of the NHO 01, and the daily dose is the ratio of the total daily time over the maximum daily time allowable at that level expressed as a percentage of sound energy.

After obtaining the daily doses, they were fitted into the technical considerations provided by the NHO 01 (Table 2), which also suggests the recommended action to reduce noise exposure, if necessary.

**Table 2:** Technical considerations and recommended action according to the daily doses found in the assessed exposure condition

Daily dose (%)	Technical consideration	Recommended Action
0 to 50	Acceptable	At least maintenance of the existing condition
50 to 80	Above the level of action	Adoption of prevention measures
80 to 100	Region of uncertainty	Adoption of preventive measures aimed at reducing the daily dose
Above 100	Above the exposure limit	Immediate adoption of corrective measures

Source: Occupational Hygiene Standard 01 (NHO 01).^5^

## RESULTS

In group 1, in the junior category, the daily dose calculation showed an exposure of 88%, or region of uncertainty (Table 3), but within the daily exposure limit of 100%. The rehearsal lasted 2 hours; however, the total real noise exposure was 7.16 minutes - which was the C_n_ value used in the referred calculation.

**Table 3:** Total exposure time, daily dose and technical consideration of group 1 and respective categories

Categories	Total time (minutes)	Daily dose (%)	Technical consideration
Junior	7.13	88	Uncertainty region
General B	33.16	423	Above exposure limit
Master	24.90	218	Above exposure limit
General A	32.49	370	Above exposure limit
Free	17.11	150	Above exposure limit

It can be seen that, in the category general B, the exposure was of 423%, the highest found in the entire survey. The duration of the rehearsal was 2 hours; however, the members’ total real exposure to noise was 33.16 minutes.

In the master category, there was an exposure of 218%. The rehearsal lasted 2 hours, but the exposure to noise was of 24.9 minutes.

With the calculation of daily dose, it was possible to verify a 370% exposure in the general A category. The total real exposure of the members to noise was 32.49 minutes in a rehearsal that lasted 2 and half hours.

In free category rehearsal, lasting 3 hours, the daily dose calculation showed an exposure of 150% in 17.11 minutes.

In group 2 (Table 4), the daily dose calculation in the adult category showed an exposure of 127%. The total real exposure to noise of members was 99.42 minutes in a 5-hour rehearsal.

**Table 4:** Total exposure time, daily dose, and technical consideration of *taiko* group 2 and respective categories

Categories	Total time (minutes)	Daily dose (%)	Technical consideration
Adults	99.42	127	Above the exposure limit
Children	16.05	17	Acceptable

The calculation of the daily dose in the child category showed and exposure of 17% in a 1-and-a-half-hour rehearsal, with real exposure to noise of 16.05 minutes.

## DISCUSSION

Tables 1 and 2, presented by the NHO 01 and adopted as a reference in this research for the assessment of occupational noise exposure, are similar to the document ‘Criteria for a Recommended Standard - Occupational Noise Exposure’,^18^ defined by the NIOSH in 1998, and to the NR15^4^ - which addresses unhealthy activities and operations. They all define the tolerance limits for continuous or intermittent noise, for each noise level, in dB. Thus, they determine the maximum daily exposure time allowable and indicate that noises above the defined values, depending on their intensity and duration, can be harmful to hearing.

From the results obtained, the junior category, in group 1, presented a daily dose in a region of uncertainty (Table 3), requiring the adoption of preventive and corrective measures aimed at reducing the dose. In this category, prevention should be enhanced, since the age range of the members varies from 6 to 18 years of age. Hearing loss related to sound exposure is a consequence of 2 fundamental aspects: the characteristics of noise and individual susceptibility. The latter is related to sex, age, and ear diseases. Age is important, as the youngest and the oldest seem to be the most susceptible.^12^

There are several noise control measures, such as: elimination, substitution, segmentation of noise source manipulation, administrative control (change in work practices and working hours, development of policies, and application of regulations aimed at worker behavior) and use of personal protective equipment (PPE).^19^ Therefore, one of the viable control measures would be the use of PPE in the form of hearing protectors, of the *plug* and/or shell type, which provide 16 dB(A) and 21 dB(A) of attenuation, respectively. The use of both protectors can attenuate noise by up to 37 dB(A).^20^

The major challenge is to convince musicians to use such a device, since the complaint that hearing protectors impair the quality of musical performance is not uncommon. In a study of 15 members of a classical music orchestra, a questionnaire on the experience of musicians with the use of the ear*plug*s was administered, in which they reported that, in addition to the PPE being uncomfortable, it affects timbre and dynamics.^21^ Still, as a second corrective measure, simple strategies such as intervals during the rehearsal and rehearsals on alternate days can be adopted, which can substantially reduce the risks of exposure to noise.^22^

In group 1, the daily doses of categories general B, master, general A, and free were 423, 218, 370, and 150, respectively; all above the 100% daily exposure limit. In such cases, immediate corrective action is recommended. It should be noted that, in the case of the master category, the susceptibility to noise in older adults seems to be increased.^12^ In the general A category, it was observed that only the exposure to 104 dB(A), which was of 8.23 minutes, exceeded the daily exposure limit of 5.95 minutes established by the NHO 01. The time of 8.23 minutes refers, almost entirely, to one song, which demonstrates that the exposure to noise is so intense that the duration of the rehearsal should be shorter than the duration of that song. In the adult category, in group 2, there was an exposure of 27% above the established value (Table 4), which also implies immediate correction measures.

To prevent hearing damage, which is potentially present in all categories of group 1 and in the adult category of group 2, there are a number of measures, among which stand out the requirement to provide hearing protectors for those exposed and hearing screening, with periodic audiometries.^23^ As the noise generated by the drums is the music itself, it becomes impracticable for the musician to move away from the source (drum), which would be the main corrective measure according to the NR9^24^ which addresses the Environmental Risk Protection Program (ERPP).

When comparing the 2 *taiko* groups, it can be seen that group 1, despite having fewer components, uses a large number of drums (each member carries his or her own drum) and, as the music itself is produced by the instruments, the sound intensity generated is higher in relation to group 2. In the latter, the number of drum models used is smaller, with choreographed movements and a musical background in playback, which makes the maximum sound intensity reached lower than that in group 1.

It was also verified that adults in both groups are already exposed to noise above the daily dose limits. On the other hand, there are participants from group 1 who play in more than one category on the same day of rehearsal, thus increasing exposure. However, it is valid to consider that other situations can help trigger and worsen hearing loss, such as those related to heredity, exposure to ototoxic substances, history of traumatic brain injury, routine use of alcoholic beverages, coffee, and smoking.^25^

In addition to the causes already mentioned, the extent of music exposure also depends on the following variables: the instrument played (drummers are more susceptible to hearing damage compared to other musicians), if playing in a group (number of musicians), the extent of the amplification, practice sites (concert halls have better acoustic treatment compared to nightclubs), as well as public participation in performances and individual musical education.^26^

Children’s rehearsals in both groups were short; however, according to the standard defined by the Brazilian Ministry of Health,27 short exposures to intense noise can also trigger hearing loss. This would justify the fact that the junior category from group 1 played less than the children from group 2, but were closer (88%) to the exposure limit (100%). Therefore, preventive and corrective changes are necessary, as previously described. For the latter category, the daily dose was 17%, which is an acceptable and safe value for maintaining the practice.

## CONCLUSIONS

Based on the NHO 01, *taiko* musicians are exposed to a daily dose beyond the acceptable limit, except in the junior (group 1) and children categories (group 2) - who presented daily exposure to noise at uncertain and acceptable levels, respectively.
